# Genome-wide imaging association study implicates functional activity and glial homeostasis of the caudate in smoking addiction

**DOI:** 10.1186/s12864-017-4124-5

**Published:** 2017-09-19

**Authors:** David C. Qian, David L. Molfese, Jennifer L. Jin, Alexander J. Titus, Yixuan He, Yafang Li, Maxime Vaissié, Humsini Viswanath, Philip R. Baldwin, Ralf Krahe, Ramiro Salas, Christopher I. Amos

**Affiliations:** 1 Department of Biomedical Data Science, Dartmouth Geisel School of Medicine, Lebanon, NH 03756 USA; 2 Menninger Department of Psychiatry and Behavioral Sciences, Baylor College of Medicine, Houston, TX 77030 USA; 3 Michael E. DeBakey Veterans Affairs Medical Center, Houston, TX 77030 USA; 4 Department of Mathematics, Dartmouth College, Hanover, NH 03755 USA; 5 Department of Epidemiology, Dartmouth Geisel School of Medicine, Lebanon, NH 03756 USA; 6 Department of Biological Sciences, Dartmouth College, Hanover, NH 03755 USA; 7 Department of Genetics, University of Texas MD Anderson Cancer Center, Houston, TX 77030 USA

**Keywords:** Functional magnetic resonance imaging, Genome-wide association studies, Smoking addiction, Caudate activity, Gene set enrichment analysis

## Abstract

**Background:**

Nearly 6 million deaths and over a half trillion dollars in healthcare costs worldwide are attributed to tobacco smoking each year. Extensive research efforts have been pursued to elucidate the molecular underpinnings of smoking addiction and facilitate cessation. In this study, we genotyped and obtained both resting state and task-based functional magnetic resonance imaging from 64 non-smokers and 42 smokers. Smokers were imaged after having smoked normally (“sated”) and after having not smoked for at least 12 h (“abstinent”).

**Results:**

While abstinent smokers did not differ from non-smokers with respect to pairwise resting state functional connectivities (RSFCs) between 12 brain regions of interest, RSFCs involving the caudate and putamen of sated smokers significantly differed from those of non-smokers (*P* < 0.01). Further analyses of caudate and putamen activity during elicited experiences of reward and disappointment show that caudate activity during reward (CR) correlated with smoking status (*P* = 0.015). Moreover, abstinent smokers with lower CR experienced greater withdrawal symptoms (*P* = 0.024), which suggests CR may be related to smoking urges. Associations between genetic variants and CR, adjusted for smoking status, were identified by genome-wide association study (GWAS). Genes containing or exhibiting caudate-specific expression regulation by these variants were enriched within Gene Ontology terms that describe cytoskeleton functions, synaptic organization, and injury response (*P* < 0.001, FDR < 0.05).

**Conclusions:**

By integrating genomic and imaging data, novel insights into potential mechanisms of caudate activation and homeostasis are revealed that may guide new directions of research toward improving our understanding of addiction pathology.

**Electronic supplementary material:**

The online version of this article (10.1186/s12864-017-4124-5) contains supplementary material, which is available to authorized users.

## Background

Tobacco smoking is the single most preventable cause of death in the world [1]. Each year, nearly 6 million deaths and over a half trillion dollars in healthcare costs worldwide are attributed to smoking [2]. The drive to better understand addiction neurobiology and devise more effective methods of smoking cessation has motivated extensive study of several brain regions. Activation of the nucleus accumbens, caudate, and prefrontal cortex is believed to mediate the reinforcing effects of nicotine in cigarettes [3]. Smokers with insula damage were shown to quit smoking more easily and remain relapse-free compared to smokers without insula damage [4]. More recently, the habenula was discovered to respond to negative events, especially in the context of abstinence when the urge to smoke is greatest [5]. However, these brain regions exhibit considerable variability in activity across individuals. In addition, little is known about genetic influences [6].

The application of genome-wide association studies (GWAS) to brain imaging data is a burgeoning new field that aims to correlate genetic factors with brain functions. Since neurologic differences across individuals are difficult to quantify, assessing intermediate phenotypes such as local brain activity via functional magnetic resonance imaging (fMRI) for GWAS has been deemed more biologically meaningful and better powered (continuous outcome variables) than standard case-control GWAS [7]. For example, GWAS paired with fMRI analysis of frontal cortex activity identified genetic variants associated with reading ability [8]; GWAS paired with dorsolateral prefrontal cortex activity identified variants associated with risk of schizophrenia [9]; and GWAS paired with volumetric estimations of subcortical structures identified variants associated with caudate, putamen, and hippocampus sizes [10]. Gene set analyses of GWAS augment biological insights by considering the joint effects of variant associations. The first such approach applied to a GWAS of brain-wide fMRI following principal components transformation proposed the importance of calcium regulation pathways in facial recognition [11].

In this study, we explored how smoking status (non-smoker, sated smoker, abstinent smoker) alters resting state as well as task-based brain signaling during reward and disappointment. Genetic variants associated with imaged brain activities were further tested for enrichment of gene sets. To the best of our knowledge, this is the first demonstration of variant-to-gene mapping for gene set analysis following GWAS that utilizes brain region-specific expression quantitative trait loci (eQTLs) instead of simply relying on variant-gene proximity [10–12]. Preliminary hypotheses regarding the relevance of implicated gene sets to brain activity during reward perception are also discussed in order to promote replication GWAS and the design of new elucidatory experiments.

## Methods

### Participants

We recruited 67 smokers (13 females, 54 males) and 86 non-smokers (49 females, 37 males) from the Houston, TX metropolitan area via fliers, newspapers, and Internet advertisements. They provided written consent for study participation. Data collection was conducted at Baylor College of Medicine and approved by its Institutional Review Board. Participants were screened to rule out non-tobacco substance dependence, pursuit of smoking cessation at the time of this study, and MRI contraindications, such as head injuries, foreign metal in body, claustrophobia, and pregnancy. Study inclusion also depended on fulfilling requirements for the personal information questionnaire (provided at least sex, age, smoking history), MRI scan (movement <2 mm), and DNA analysis (genotyping call rate > 95%). Smokers were scanned two separate times by MRI, once after they had been instructed to smoke *ad libitum* (“sated”) and a second time after they refrained from smoking for at least 12 h (“abstinent”). Smokers completed the Shiffman-Jarvik Withdrawal Questionnaire (SJWQ) as well, a self-reported measure of tobacco dependence and withdrawal [13]. As compliance assurance, we excluded smokers whose abstinent levels of exhaled carbon monoxide (Micro+Smokerlyzer, Bedfont Scientific) did not decrease by at least 40% compared to corresponding sated levels. In total, 119 participants with imaging data who completed the questionnaire and 116 participants with genotype data who completed the questionnaire were retained for further analysis (Table 1). Genotype-fMRI association testing was performed on the 106 participants with both types of data.

**Table 1:** Participant characteristics

	Non-smokers	Smokers		*P*		
		Sated	Abstinent	NS vs. Sat	NS vs. Abs	Sat vs. Abs
Male	37%	65%		2.65 × 10^−3^		
Age	30.9 (11.3)	42.4 (11.7)		1.80 × 10^−6^		
Cigarettes per day		15.9 (7.0)				
Years of smoking		26.2 (12.7)				
Lifetime pack-years		22.1 (16.2)				
Carbon monoxide^a^		21.9 (12.6)	10.2 (6.8)			3.95 × 10^−9^
FTND^a^		5.1 (2.1)	5.1 (2.0)			0.946
PANAS^a^		−7.4 (10.2)	−7.5 (9.5)			0.976
SJWQ^a^		3.4 (0.7)	3.9 (1.0)			1.77 × 10^−3^
RSFC						
Caudate-amygdala	0.148 (0.102)	−0.539 (0.146)	0.361 (0.149)	3.40 × 10^−4^	0.277	1.38 × 10^−4^
Caudate-mPFC	−0.241 (0.125)	0.356 (0.117)	−0.001 (0.157)	9.08 × 10^−4^	0.265	0.099
Caudate-sPFC	−0.224 (0.118)	0.309 (0.144)	0.023 (0.145)	6.48 × 10^−3^	0.221	0.198
Caudate-GP	0.211 (0.118)	−0.294 (0.138)	−0.020 (0.154)	7.91 × 10^−3^	0.269	0.225
Putamen-mPFC	−0.261 (0.124)	0.244 (0.120)	0.159 (0.159)	4.93 × 10^−3^	0.054	0.696
Caudate activity, during						
Reward	0.186 (0.847)	−0.317 (1.070)	0.002 (1.100)	0.015	0.358	
Disappointment	0.038 (0.984)	−0.139 (0.975)	0.072 (1.056)	0.376	0.868	
Putamen activity, during						
Reward	0.112 (0.981)	−0.067 (1.062)	−0.114 (0.973)	0.393	0.244	
Disappointment	−0.012 (1.040)	0.002 (0.885)	0.021 (1.059)	0.957	0.872	

Mean participant characteristics are displayed with standard errors in parentheses. Caudate and putamen fMRI activities during reward and disappointment are presented as *z*-scores. The sample sizes for each comparison differ depending on type of data collected, as discussed in the Methods section: non-smokers range from *N* = 64 to *N* = 67 and smokers range from *N* = 42 to *N* = 52. ^a^ denotes comparison using paired *t*-test; all other comparisons were performed using unpaired *t*-tests and *χ* ^2^ tests for continuous and categorical variables, respectively. *Abs* abstinent smokers; *FTND* Fagerstrom Test for Nicotine Dependence, *GP* globus pallidus, *mPFC* medial prefrontal cortex, *NS* non-smokers, *PANAS* Positive And Negative Affect Scale, *RSFC* resting state functional connectivity, *Sat* Sated smokers, *SJWQ* Shiffman-Jarvik Withdrawal Questionnaire, *sPFC* superior prefrontal cortex

### MRI procedure

All MRI data were collected using 3 T MAGNETOM Trio scanners (Siemens). Each MRI session began with a magnetization-prepared rapid gradient echo (MPRAGE) structural scan (160 axial slices, 1 × 1 × 1 mm voxels, TE = 2.66 ms, TR = 1.2 s, flip angle = 12°, 256 × 256 matrix). Five minutes of resting state functional connectivity (3.4 × 3.4 × 4.0 mm voxels, TE = 40 ms, TR = 2.0 s, flip angle = 90°) followed by 20 min of regional activity during elicited reward and disappointment (echo-planar imaging, 2 × 2 × 2 mm voxels, TE = 40 ms, TR = 2.0 s, flip angle = 90°) were then measured as previously described [14, 15].

### Resting state functional connectivity (RSFC)

RSFC captured activity correlation between brain regions [16], during which participants had been instructed to simply “let your mind wander” for 5 min. They were free to keep eyes open or closed, as the strength of measured RSFC is negligibly affected by these states [17]. Acquired images were preprocessed using the software Statistical Parametric Mapping 8 [18]. Preprocessing included realignment, co-registration to the mean image, segmentation, normalization to the standard Montreal Neurological Institute (MNI) atlas [19], and full-width at half-maximum Gaussian smoothing with a 6 mm kernel. White matter and cerebral spinal fluid were used as possible confounders, and a band pass filter (0.008–0.09 Hz) was applied. With imaging coordinates expressed as “(translational parameters x, y, z; rotational parameters roll, pitch, yaw)”, we selected the amygdala (21.8, 3.0, −12.2; −25.7, 1.9, −12.3), anterior cingulate cortex (ACC; 4.0, −30.4, 18.0; −8.5, −32.0, 19.8), caudate (10.8, −6.9, 11.6; −14.1, −8.0, 11.8), globus pallidus (GP; 17.7, 5.0, 4.2; −21.2, 4.8, 4.2), habenula (3, −22, 4; −3, −22, 4), insula (32.5, −4.4, 6.1; −36.6, −3.9, 5.1), nucleus accumbens (NAcc; 10.6, −5.4, −6.5; −11.2, −7.9, −4.3), prefrontal cortex subdivisions (PFC; inferior 40.1, −21.9, 10.3; −44.7, −21.9, 11.3 | medial 31.2, −26.2, 35.7; −35.5, −27.0, 34.4 | superior 16.9, −29.3, 41.2; −20.4, −25.9, 42.2), putamen (22.1, −0.6, 5.3; −26.0, −1.8, 5.3), and supplementary motor area (SMA 5.3, 0.2, 65.4; −8.6, 4.8, 65.9) as regions of interest (ROIs) for investigation, given their previously implicated roles in addiction and impulsivity [14, 20–22].

A center coordinate for each of the left and right habenula was manually identified. Three cubic millimeter seeds were created around the central coordinates for the left and right habenula of every participant. This was done because the habenula is a very small region that necessitates manual identification, while all other ROIs were drawn in the software Analysis of Functional NeuroImages (AFNI) [23]. Finally, ROIs were input for processing by the Matlab toolbox CONN [24]. Fisher-transformed correlation coefficients from these analyses were recorded as RSFCs. Significant differences in RSFCs for ROI pairs involving the caudate and putamen were detected between non-smokers and smokers by unpaired *t*-test comparisons (Additional file 1: Figure S1 and Table S1). Although RSFCs are expected to be dependent, this dependence structure is unknown. The *P*-value cutoff for multiple RSFC comparison tests with arbitrary dependence was computed by the Benjamini-Hochberg-Yuketieli (BHY) procedure [25] to be 0.0104.

### Task-based reward and disappointment

We then examined caudate and putamen activities while participants performed a task that elicits experiences of reward and disappointment. As previously described [14, 15], participants were conditioned to anticipate the delivery of 1 ml Crystal Light juice (individual flavor preference made beforehand) from a Standard Infuse/Withdraw 33 Twin Syringe Pump (Harvard Apparatus) following a yellow circular light visual cue presented by ePrime software (Psychology Software Tools) over 55 consecutive iterations. Juice delivery lasted 1 s. Normally, juice delivery occurred 6 s after the visual cue (expected “reward”). In the last few iterations, between 6 and 12 juice deliveries were randomly delayed by an additional 4 s (unexpected “disappointment”) in a 1:2 ratio with normal events to prevent rapid learning of paradigm change [26]. We originally intended to also ascertain measurements from the habenula, but they did not pass quality control standards due to the region’s small size.

Raw DICOM format images were converted to NIfTI format, and anatomical and functional images were processed using the standard AFNI processing stream [23]. Voxels above an outlier threshold of 15% were rejected by 3dToutcount. The remaining functional data were corrected for slice-time acquisition using 3dTshift. 3 × 3 × 3 mm voxels were aligned to the first image and measured for motion (3dvolreg), registered to the high resolution MPRAGE, and transformed to MNI space using a single spatial transform (@auto_tlrc, 3dAllineate). Data were smoothed using a 4.5 mm kernel in 3dmerge and subjected to deconvolution by generalized linear model (GLM) regression in 3dDeconvolve. The GLM design matrix included regressors for motion parameters, linear, quadratic, and cubic trends, and four stimulus conditions (visual cue, juice reward, absence of expected juice reward, late juice reward) [27]. Cubic spline interpolation corrects for image-by-image movement and relative time differences in acquisition of individual brain slice data. In general, use of higher order terms better fits temporal and spatial jitter in image collection and allows for better resolution of individual stimulus types, as motion- and time-corrected whole brain images are reconstituted from a stack of individual slices.

### Genome-wide association study

**Fig. 1:**
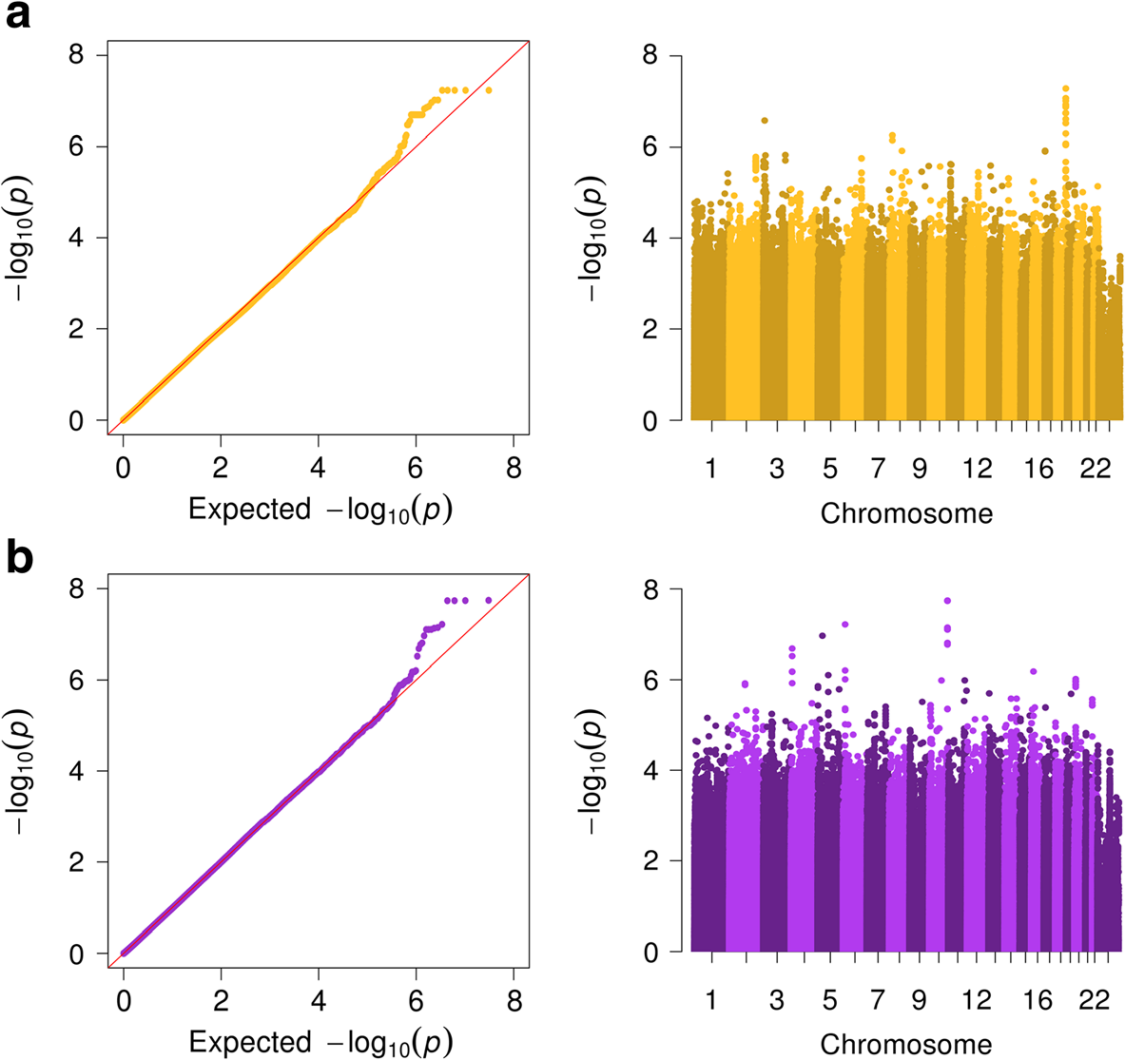
Quantile-quantile and Manhattan plots of GWAS results. With adjustment for sated smoker status and population stratification, allelic dosages of germline variants were linearly regressed on (**a**) caudate activity during reward and (**b**) putamen activity during disappointment. The negative logarithms (base 10) of observed *P*-values were plotted in relation to those of expected *P*-values and chromosomal position

We genotyped 200 ng DNA samples from buccal swabs using the HumanOmniExpress-12 v1.1 BeadChip and Infinium HD Ultra Assay protocol (Illumina). Following exclusion of single nucleotide polymorphisms and indels (collectively referred to as “markers”) with minor allele frequency less than 5%, genotyping rate less than 90%, and deviation from Hardy-Weinberg equilibrium at the 0.0001 significance level, 621,854 genetic markers were imputed to the most recent panel of the 1000 Genomes Project (Phase 3 integrated release, October 2014) [28] with haplotype phasing by SHAPEIT [29] using IMPUTE v2.3.1 [30]. Marker imputations were filtered for having information measure greater than 0.9. Imputed allelic dosages were then tested for additive associations with fMRI activities of the caudate and putamen during reward and disappointment (Fig. 1) using linear regression in SNPTEST v2.5.2 [31]. These GWAS were adjusted for sated smoker status and population stratification, as represented by the top 3 principal components of non-imputed genotype data derived using PLINK v1.9 [32].

### Gene set enrichment

Gene-marker correspondences were established based on whether independently associated variants reside within genes, or whether nominally significant variants influence tissue-specific gene expression. A variant was deemed to be independently associated if it displayed both *P*_GWAS_ < 0.05 and *P*_conditional_ < 0.001 from stepwise-selection conditional analysis by the software Genome-wide Complex Trait Analysis (GCTA) [33]. We applied this method to avoid identifying many strongly associated markers in linkage disequilibrium. A nominally significant variant was deemed to influence gene expression if it displayed *P*_GWAS_ < 0.05 and has been considered an eQTL in non-diseased caudate or putamen by the Genotype-Tissue Expression (GTEx) project v6 [34]. Choices for *P*_GWAS_ and *P*_conditional_ cutoffs were motivated by the desire to include as many variants as possible, within biologic and biostatistical reason. *P*_GWAS_ < 0.05 is commonly implemented when downstream variant filters are sufficiently stringent [35–38]. In this case, variants must be intragenic independent associations or tissue-specific eQTLs. *P*_conditional_ < 0.001 is the most relaxed cutoff that GCTA endorses [33].

Upon assigning each gene the largest –log_10_ *P*_GWAS_ of the variant(s) from which it was mapped, we then conducted Gene Set Enrichment Analysis (GSEA) using curated pathways from the Molecular Signatures Database (MSigDB) [39] as well as biologic processes, molecular functions, and cellular components from the Gene Ontology (GO) project [40]. In brief, GSEA assesses the tendency of genes in a gene set to appear at the top/bottom of a ranked list (i.e. GWAS results) by computing the gene set’s enrichment score (ES) as the maximum deviation of a running-sum statistic from zero. The significance of an ES is determined from the null distribution of ESs generated by 10,000 permutations of pathway labels. However, ES does not account for gene set size (larger gene sets are more likely to contain high ranking genes) or multiple testing (greater quantity of tested gene sets is more likely to spuriously yield high ESs). To remedy these biases, gene rank order is permuted 10,000 times and the ES for every gene set is recalculated accordingly. Each gene set’s observed ES and permutation-based ESs are then divided by the mean ES across permutations to produce normalized ESs (NESs), but only using the positive observed ESs and permutation-based ESs. Negative ESs characterize gene sets overrepresented at the bottom of GWAS results, which are not of interest to our analysis. False discovery rate (FDR) is the fraction of permutation-based NESs that exceed the observed NES [39].

## Results

Descriptive statistics of participants are outlined in Table 1. Not surprisingly, smokers exhaled less carbon monoxide and had greater smoking cravings when abstinent compared to when sated. Although smokers were more likely to be male and older than non-smokers, incorporation of sex and age as variables in linear regression revealed no significant impact on RSFCs or task-based fMRI activities (*P* > 0.05). Among smokers, trends between lifetime pack-years and RSFCs or task-based fMRI activities do not survive multiple testing correction using BHY (*P* > 0.01). However, categorical smoker status did correlate with some imaging measurements. The RSFCs of sated smokers between caudate and amygdala, between caudate and mPFC, between caudate and sPFC, between caudate and GP, and between putamen and mPFC significantly differed from those of non-smokers (Additional file 1: Figure S1 and Table 1). Abstinent smokers did not differ from non-smokers for all 66 RSFCs between the 12 ROIs (also *P* > 0.01, Additional file 1: Figure S1). These findings corroborate previous studies of frontostriatal circuit changes due to pleasure-seeking behavior, such as smoking and drug use [41–43].

Given the suspected involvement of striatum components, the caudate and putamen, in modulating brain signaling at rest with respect to smoking, we further analyzed their activities when participants experienced reward and disappointment. Distributions of GWAS *P*-values for caudate activity during disappointment and putamen activity during reward did not positively deviate from the null, as seen in quantile-quantile plots (Additional file 1: Figure S2). In contrast, the significance of many genetic associations with caudate activity during reward (CR) and putamen activity during disappointment (PD) surpassed what would be expected by chance alone (Fig. 1). CR also differed by smoking status (*P* = 0.015) and was negatively associated with SJWQ withdrawal symptoms among abstinent smokers (*P* = 0.024); PD did not display such relationships (Table 1 and Additional file 1: Figure S3). We therefore focused subsequent analyses only on CR.

Ranked results from GWAS of CR are presented in Additional file 1: Table S2. The top locus, chromosome 18q22.2, is not near any gene. It is over 50 kilobases downstream of *CCDC102B* (encoding a poorly annotated coiled-coil protein) and over 140 kilobases upstream of *DOK6* (encoding an intracellular adaptor of the Ret signaling cascade). In addition, none of the associated markers in this locus are caudate-specific eQTLs of genes within 1 megabase according to GTEx. The sentinel marker (rs11718289, *P* = 2.95 × 10^−7^) of the second most significant locus, chromosome 3p25.3, is an overlapping intron variant within both *CAV3* (caveolin 3) and *OXTR* (oxytocin receptor). The caveolin family of proteins organizes caveolae, which are flask-shaped plasma membrane invaginations that participate in endocytosis [44], calcium homeostasis [45], compartmentation of membrane-bound receptors [46], and cytoskeleton anchoring [47]. In the brain, caveolins 1 and 2 are more commonly expressed in endothelial cells, while caveolin 3 predominates in astrocytes [48]. Oxytocin receptors transduce signaling by the hormone and neurotransmitter oxytocin. Oxytocin activation of fMRI-interrogated ROIs in the mesolimbic reward pathway has been shown to influence sexual, social, and addictive behavior [49].

With respect to cumulative genetic effects, genes that contain variants independently associated with CR and/or that exhibit expression regulation by variants nominally associated with CR (Additional file 1: Table S3) were tested for enrichment of MSigDB and GO gene sets using GSEA [39]. None of the MSigDB gene sets yielded *P* < 0.05 and FDR < 0.05. Since the curated gene sets of MSigDB tend to represent well-studied pathways, lack of results from this database reiterates the consensus that genetic mechanisms underlying brain development and signaling remain poorly understood, especially relative to those of other tissues [50]. For this reason, GO gene sets were also included in our analysis. They describe broader collections of genes that share molecular functions, co-localize within cells, or participate together in biologic processes [40]. So even though GO gene sets do not facilitate the implication of precisely defined pathways, they are still useful for guiding univariate gene associations toward the identification of organized biologic themes.

Of the 4726 gene sets from MSigDB and 23,462 gene sets from GO (as of 24 January 2016), GSEA highlighted 11 GO gene sets that exhibit FDR < 0.05 with appropriate adjustment for gene size, gene set size, and multiple testing (Table 2 and Additional file 1: Table S4). Three main themes emerged: electrical signal transmission (pathway #s 6, 8, and 9 in Table 2), cellular response to biochemical stimuli (pathway #s 2, 3, and 4 in Table 2), and cytoskeleton organization (pathways #s 1, 7, and 11 in Table 2). Perfusion and carbohydrate metabolism (pathway #s 5 and 10 in Table 2) may be genuine findings, as well as indicators of experimental reliability. fMRI assesses oxygenated blood flow as a proxy for regional activity in the brain, which primarily depends on glucose for energy.

**Table 2:** Gene set enrichment analysis results

Gene set	P	FDR
1. CELL LEADING EDGE (CC, GO:0031252)	<0.0001	0.0202
2. CELLULAR RESPONSE TO ENDOGENOUS STIMULUS (BP, GO:0071495)	0.0001	0.0226
3. RESPONSE TO WOUNDING (BP, GO:0009611)	<0.0001	0.0242
4. ENZYME LINKED RECEPTOR PROTEIN SIGNALING (BP, GO:0007167)	0.0001	0.0277
5. BLOOD CIRCULATION (BP, GO:0008015)	0.0006	0.0295
6. REGULATION OF ION TRANSMEMBRANE TRANSPORT (BP, GO:0034765)	0.0006	0.0361
7. CYTOSKELETAL PROTEIN BINDING (MF, GO:0008092)	<0.0001	0.0367
8. SYNAPSE ORGANIZATION (BP, GO:0050808)	0.0005	0.0373
9. DENDRITE (CC, GO:0030425)	0.0002	0.0389
10. CARBOHYDRATE HOMEOSTASIS (BP, GO:0033500)	0.0001	0.0434
11. NEURON PROJECTION DEVELOPMENT (BP, GO:0031175)	0.0003	0.0467

Gene Ontology identifiers are displayed in parentheses. Types of Gene Ontology gene sets: *BP* biologic process, *CC* cellular component, *MF* molecular function

## Discussion

The caudate is an important component of the brain’s reward circuitry [51]. In the present study, caudate RSFCs differed between sated smokers and non-smokers. No RSFC differences were observed between abstinent smokers and non-smokers. Reward events also triggered caudate activities that exhibit significant association with genetic variants, independent of smoking status. The top gene-based locus from GWAS of CR is within *CAV3*, a gene expressed in astrocytes and a key driver of enrichment for multiple GO gene sets (Additional file 1: Table S4). *OXTR* contains the locus of interest as well, but GSEA results revealed less extensive representation by *OXTR*. Significant variants jointly tended to reside within or influence the expression of genes that contribute to electrical signal transmission, cellular response to biochemical stimuli, and cytoskeleton organization. These GO findings are each discussed in turn. They strongly implicate the versatile functions of astrocytes in maintaining central nervous system (CNS) homeostasis.

Statistical enrichment of CR-associated genetic variations within gene sets that involve electrical signal transmission is not entirely surprising. When ions cross the membrane of a neuron (pathway #6 in Table 2), action potentials are generated and travel along the length of the neuron (pathway #11 in Table 2). Axon terminus depolarization then triggers neurotransmitter release into synapses (pathway #8 in Table 2) between the neuron at hand and message-receiving dendrites of downstream neurons (pathway #9 in Table 2). Following neurotransmitter-gated ion channel opening, electrical signals continue to propagate. Using an assembly of membrane proteins including caveolin-3, astrocytes also tune neuronal excitability by regulating ion concentrations in synapses and the extracellular space [52, 53].

Cigarette smoke consists of many compounds that exert toxic effects on the brain. Genetically-driven variation in how brain cells respond to these effects (pathway #s 2, 3, and 4 in Table 2) is therefore likely to influence individual CR as well. First, cigarette smoke damages and increases the permeability of the blood-brain barrier (BBB) [54]. Second, compromised integrity of the BBB allows reactive radicals from cigarette smoke to more profoundly inflict inflammation and oxidative stress within the CNS. The ensuing secretion of cytokines, such as interferon γ, tumor necrosis factor α, and interleukin 6, has been shown to promote not only greater leukocyte infiltration from the systemic circulation [55], but also inappropriate attack on brain cells by resident microglia [56]. Third, oxidative stress can lead to impaired respiration in mitochondria and activate c-Jun N-terminal kinases, both mediators of apoptosis, to induce neuronal atrophy, depletion of dendritic spines, and demyelination [57–59].

The final theme among our pathway results, cytoskeleton organization, unifies all of the aforementioned biologic processes. Interactions between actin filaments and microtubules along with their protrusion-polymerization dynamics (pathway #s 1 and 7 in Table 2) give rise to the dendrite and axon outgrowths (pathway #11 in Table 2) that characterize mature neurons [60]. Similarly, actin filament turnover drives the wrapping action of oligodendrocyte leading edges around axons to form multilamellar myelin sheaths [61], which increase electrical impulse speed by facilitating saltatory conduction (pathway #6 in Table 2). Compared to the morphology of most other cell types, CNS cells are highly distorted and require an elaborate intracellular transport system to distribute organelles, signaling molecules, and membrane proteins across vast distances. Microtubules and their associated motor proteins of the kinesin and dynein families fulfill this need [62]. For example, neurotransmitters synthesized in the soma are packaged into vesicles and carried to the pre-synaptic membrane (pathway #8 in Table 2) on microtubule tracks. Corresponding neurotransmitter receptors arrive at the post-synaptic dendrite membrane (pathway #9 in Table 2) in an analogous fashion.

Cytoskeleton organization also plays a major role in the sequelae of neuronal injury, one of the many harmful consequences of cigarette smoking. Unlike neurons of the peripheral nervous system, those of the CNS have minimal regenerative capacity. To the extent that CNS neurons can sprout up to only a few millimeters following injury (pathway #s 2, 3, and 4 in Table 2), successful formation of new growth cones depends on a large initial calcium influx. This event then triggers cytoskeleton de-polymerization, plasma membrane collapse to seal damaged areas, eventual efflux and sequestration of calcium, cytoskeleton re-polymerization, recruitment of organelles and vesicles on microtubule tracks to replenish damaged areas, and finally mechanical force generation by actin filaments to initiate new growth cones [63]. Astrocytes impede regeneration by rapidly mobilizing their intermediate filament cytoskeleton upon injury detection to replace damaged sites with glial scars and secrete inhibitory growth factors [64].

Taken together, caudate activity is independently associated with smoking status and with germline variants linked to electrical signal transmission, cellular response to injury, and cytoskeleton organization. CR was found to be lower among sated smokers compared to non-smokers, while abstinent smokers with lower CR were prone to display greater withdrawal symptoms. Smoking may temporarily induce individuals to derive less reward from otherwise pleasurable experiences through curtailing caudate activity [65]. Additional smoking for supplemental pleasure then perpetuates addiction. Even during abstinence from smoking, addiction may be propelled by elevated craving in the setting of inherently diminished CR. Therefore uncovering the genetic basis of caudate activity variation is important to better understand the individual-level factors responsible for substance addiction and pave the foundation toward ever-more promising interventions.

This study is limited by sample size and availability of brain region-specific bioinformatic resources. Although the sample size would be considered small for most case-control GWAS, it is similar to those of previously conducted fMRI GWAS given the expense and time associated with imaging acquisition [8, 9]. Future expansion of the Enhancing Neuro Imaging Genetics through Meta-Analysis (ENIGMA) Network to include smoking and drug use working groups may facilitate the necessary collaboration to dramatically boost sample size, as in the consortium’s GWAS of regional volumes, surface areas, and cortical thickness [66].

To our knowledge, we are demonstrating the first use of caudate-specific eQTL data from GTEx; previous gene set analyses of fMRI GWAS simply mapped variants to genes based on proximity rather than correlation with gene expression [10–12]. As a form of sensitivity assessment, we further compared the utility of using biologically guided eQTLs versus just variant proximity to nominate genes for GSEA. *P*-values of independently associated variants from GWAS of CR were assigned to genes within 5 kb, 20 kb, 50 kb, 100 kb, 500 kb, and 1 Mb. Genes were then ranked by −log_10_ *P*-value and processed through GSEA as was done for our main analysis. Only the 20 kb window yielded 3 gene sets with FDR < 0.05; they happened to be pathway #s 2, 4, and 11 in Table 2. Restricting to 5 kb was too narrow. Dramatically widening the window likely introduced too much noise. Whereas GTEx helps our analysis by listing caudate-specific eQTL genes within 1 Mb [34], mapping variants indiscriminately to every gene within 50 kb to 1 Mb identifies too many unrelated genes and taints gene set enrichment. Interestingly, our finding corroborates the often cited 20 kb window as an ideal empirical mapping range for pathway analysis of GWAS [67].

In general, epigenetic and molecular pathway-based annotations for the brain are still largely in development. No documented *cis*-eQTL of the caudate or any other brain region in GTEx resides within the top locus from our GWAS of CR (chromosome 18q22.2). If polymorphisms in this locus are truly related to CNS expression levels of a gene, it may represent a crucial missed finding not only by itself, but also as part of GSEA for implicating entire biologic processes. As was done for the chromosome 8q24 locus, which is a 1.2-megabase gene desert containing variants that affect risk for multiple cancers through long-distance interaction with *MYC*, chromatin conformation capture has the potential to clarify functional significance [68] of the top locus in the present GWAS. Highlighted gene sets and their CR-associated gene constituents (Additional file 1: Tables S3 and S4) may also guide new directions of research in cell and animal models, for example using systematic multiplex mutagenesis [69].

## Conclusions

Tobacco smoking significantly contributes to worldwide morbidity and mortality. Addiction is believed to be partially mediated by smoking-induced changes to the caudate and other individual-specific factors. Here, our findings suggest that caudate signaling with the amygdala, prefrontal cortex, and globus pallidus differs between sated smokers and non-smokers at rest, but not between abstinent smokers and non-smokers at rest. In the context of rewarding events, caudate activity is lowest among sated smokers and may also be affected by genetic variants that influence pathways of glial homeostasis. These insights on the interplay of caudate signaling, smoking pathophysiology, and molecular genetic influences are likely to motivate new ideas for future studies as well as the design of more effective smoking interventions.

## Additional file

Additional file 1: Figure S1. Resting state functional connectivity (RSFC) among 12 brain regions. Rows and columns indicate the regions that comprise pairs for which RSFCs were computed. White, dark-gray, and light-gray bars represent average *z*-scores from magnetic resonance imaging measurements for non-smokers, sated smokers, and abstinent smokers, respectively, with standard errors marked above. All tickmarks are spaced 0.1 apart. Asterisks highlight differences that are significant at the 0.01 level. ACC, anterior cingulate cortex; GP, globus pallidus; NAcc, nucleus accumbens; iPFC, inferior prefrontal cortex; mPFC, medial prefrontal cortex; sPFC, superior prefrontal cortex; SMA, supplementary motor area. **Figure S2.** Quantile-quantile plots of GWAS results. With adjustment for smoker status and population stratification, allelic dosages of germline variants were linearly regressed on (A) caudate activity during disappointment, and (B) putamen activity during reward. The negative logarithms (base 10) of observed *P*-values were plotted in relation to those of expected *P*-values. **Figure S3.** Abstinence-associated withdrawal severity versus caudate activity during reward (CR) and putamen activity during disappointment (PD). After smokers had refrained from smoking for at least 12 h, the severity of their withdrawal symptoms was assessed using the Shiffman-Jarvik Withdrawal Questionnaire (SJWQ) and plotted in relation to (A) CR and (B) PD. As in Table 1, CR and PD are presented as *z*-scores following normalization that also took into account values from sated smokers and non-smokers. **Table S1.** Resting state functional connectivity (RSFC) differences among non-smokers, sated smokers, and abstinent smokers. Comparisons were performed using unpaired *t*-tests. ACC, anterior cingulate cortex; GP, globus pallidus; NAcc, nucleus accumbens; iPFC, inferior prefrontal cortex; mPFC, medial prefrontal cortex; sPFC, superior prefrontal cortex; SMA, supplementary motor area. **Table S2.** Top 5000 results from GWAS of caudate activity during reward. With adjustment for smoker status and population stratification, linear regression using allelic dosages of germline variants was performed. b, effect size from linear regression; MAF, minor allele frequency; s.e., standard error of effect size from linear regression. **Table S3.** Genes that contain or exhibit expression regulation by variants highlighted in GWAS of caudate activity during reward. Germline variants with *P*_GWAS_ < 0.05 found to be either independent associations by conditional analysis (*P*_conditional_ < 0.001) or caudate-specific expression quantitative trait loci (eQTL false discovery rate < 0.05) were identified along with their affiliated genes. All eQTL attributes were imported from the Genotype-Tissue Expression project. **Table S4.** Gene set enrichment analysis results. Gene sets consisting of genes that tend to appear near the top of GWAS results were identified using the correspondences between genes and GWAS *P*-values established in **Table S3.** The most significant *P*-value of a variant contained within each gene or deemed to affect its expression is also listed in parentheses. ES, enrichment score; FDR, false discovery rate; NES, normalized enrichment score. (ZIP 2029 kb)

## Abbreviations

ACC: Anterior cingulate cortex
AFNI: Analysis of Functional NeuroImages
BBB: Blood-brain barrier
BHY: Benjamini-Hochberg-Yuketieli procedure
CNS: Central nervous system
CR: Caudate activity during reward
eQTL: expression quantitative trait locus
ES: Enrichment score
FDR: False discovery rate
fMRI: Functional magnetic resonance imaging
GCTA: Genome-wide Complex Trait Analysis
GLM: Generalized linear model
GO: Gene Ontology
GP: Globus pallidus
GSEA: Gene Set Enrichment Analysis
GTEx: Genotype-Tissue Expression project
GWAS: Genome-wide association study
MNI: Montreal Neurological Institute
MPRAGE: Magnetization prepared rapid gradient echo
MRI: Magnetic resonance imaging
MSigDB: Molecular Signatures Database
NAcc: Nucleus accumbens
NES: Normalized enrichment score
PD: Putamen activity during disappointment
PFC: Prefrontal cortex
ROI: Region of interest
RSFC: Resting state functional connectivity
SJWQ: Shiffman-Jarvik Withdrawal Questionnare
SMA: Supplementary motor area
TE: Echo time
TR: Repetition time
