# Supplementary figures and images for: Genome-wide imaging association study implicates functional activity and glial homeostasis of the caudate in smoking addiction
Source: BMC Genomics. 2017 Sep 19;18:740. doi: 10.1186/s12864-017-4124-5 (PMC5605997; doi:10.1186/s12864-017-4124-5)

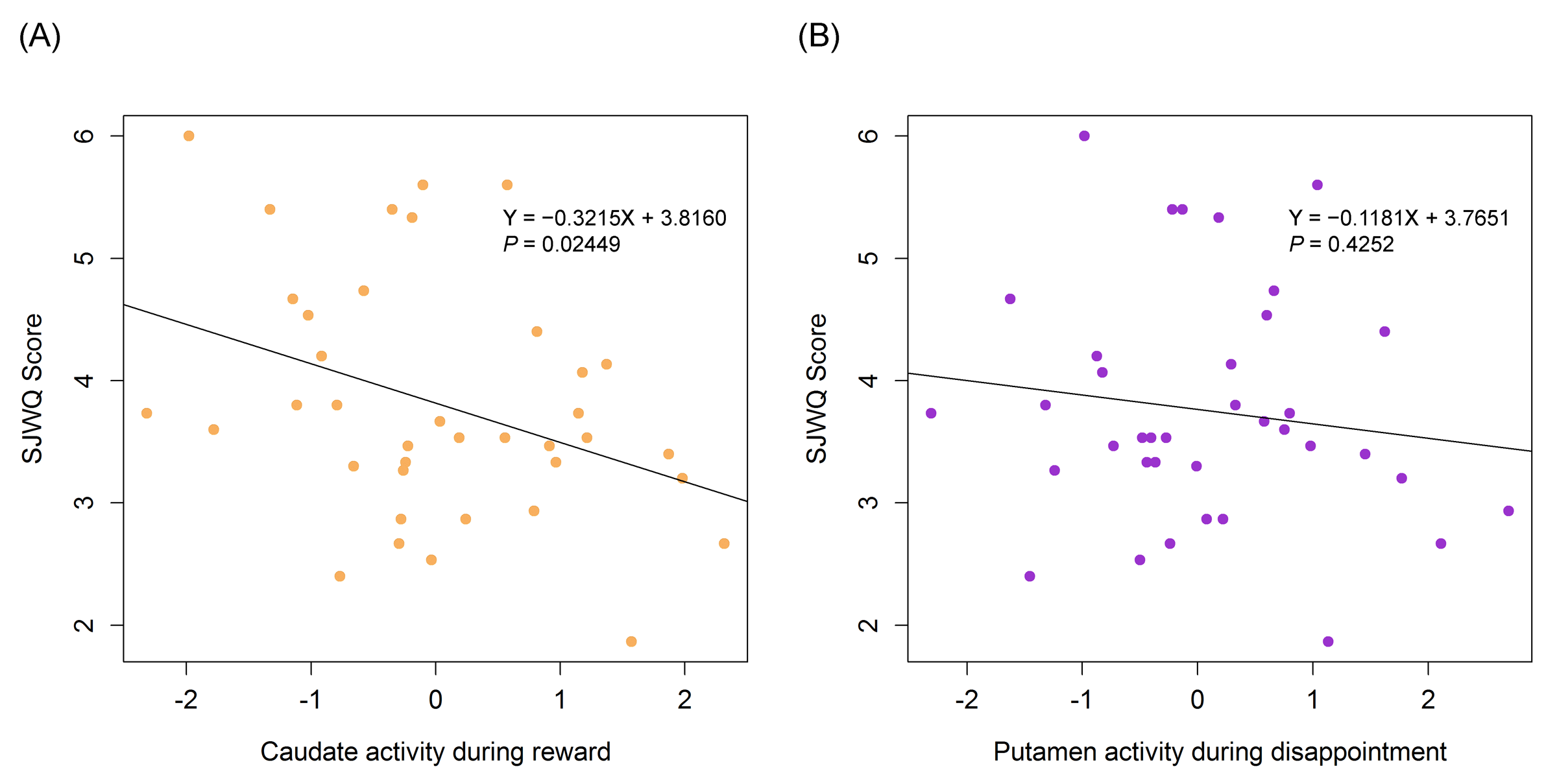

Supplement: Additional file 1: Figure S1. — Resting state functional connectivity (RSFC) among 12 brain regions. Rows and columns indicate the regions that comprise pairs for which RSFCs were computed. White, dark-gray, and light-gray bars represent average z-scores from magnetic resonance imaging measurements for non-smokers, sated smokers, and abstinent smokers, respectively, with standard errors marked above. All tickmarks are spaced 0.1 apart. Asterisks highlight differences that are significant at the 0.01 level. ACC, anterior cingulate cortex; GP, globus pallidus; NAcc, nucleus accumbens; iPFC, inferior prefrontal cortex; mPFC, medial prefrontal cortex; sPFC, superior prefrontal cortex; SMA, supplementary motor area. Figure S2. Quantile-quantile plots of GWAS results. With adjustment for smoker status and population stratification, allelic dosages of germline variants were linearly regressed on (A) caudate activity during disappointment, and (B) putamen activity during reward. The negative logarithms (base 10) of observed P-values were plotted in relation to those of expected P-values. Figure S3. Abstinence-associated withdrawal severity versus caudate activity during reward (CR) and putamen activity during disappointment (PD). After smokers had refrained from smoking for at least 12 h, the severity of their withdrawal symptoms was assessed using the Shiffman-Jarvik Withdrawal Questionnaire (SJWQ) and plotted in relation to (A) CR and (B) PD. As in Table 1, CR and PD are presented as z-scores following normalization that also took into account values from sated smokers and non-smokers. Table S1. Resting state functional connectivity (RSFC) differences among non-smokers, sated smokers, and abstinent smokers. Comparisons were performed using unpaired t-tests. ACC, anterior cingulate cortex; GP, globus pallidus; NAcc, nucleus accumbens; iPFC, inferior prefrontal cortex; mPFC, medial prefrontal cortex; sPFC, superior prefrontal cortex; SMA, supplementary motor area. Table S2. Top 5000 results from GWAS of c [file 12864_2017_4124_MOESM1_ESM.zip › Figure S3. SJWQ vs. CR and PD.tiff]

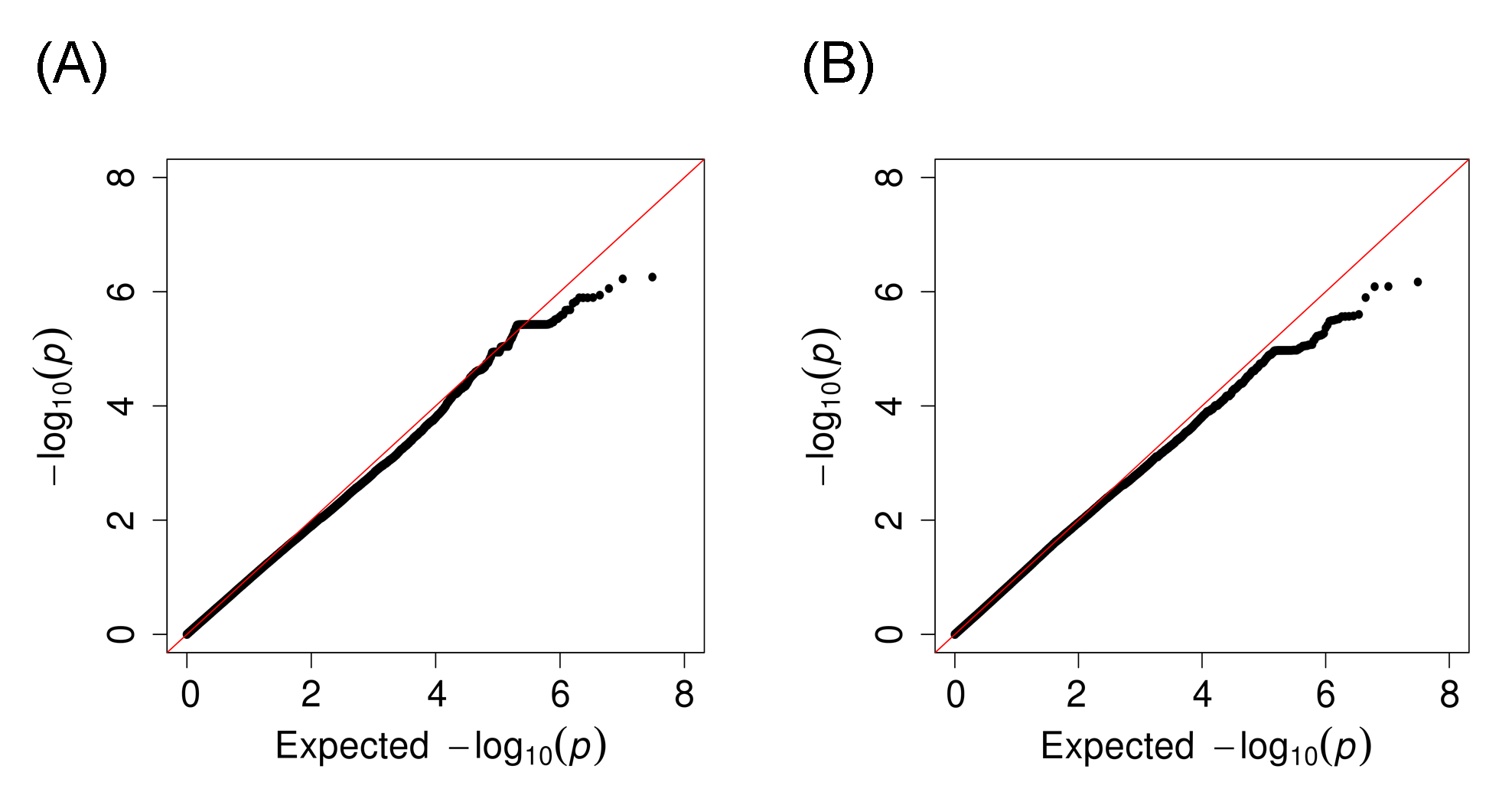

Supplement: Additional file 1: Figure S1. — Resting state functional connectivity (RSFC) among 12 brain regions. Rows and columns indicate the regions that comprise pairs for which RSFCs were computed. White, dark-gray, and light-gray bars represent average z-scores from magnetic resonance imaging measurements for non-smokers, sated smokers, and abstinent smokers, respectively, with standard errors marked above. All tickmarks are spaced 0.1 apart. Asterisks highlight differences that are significant at the 0.01 level. ACC, anterior cingulate cortex; GP, globus pallidus; NAcc, nucleus accumbens; iPFC, inferior prefrontal cortex; mPFC, medial prefrontal cortex; sPFC, superior prefrontal cortex; SMA, supplementary motor area. Figure S2. Quantile-quantile plots of GWAS results. With adjustment for smoker status and population stratification, allelic dosages of germline variants were linearly regressed on (A) caudate activity during disappointment, and (B) putamen activity during reward. The negative logarithms (base 10) of observed P-values were plotted in relation to those of expected P-values. Figure S3. Abstinence-associated withdrawal severity versus caudate activity during reward (CR) and putamen activity during disappointment (PD). After smokers had refrained from smoking for at least 12 h, the severity of their withdrawal symptoms was assessed using the Shiffman-Jarvik Withdrawal Questionnaire (SJWQ) and plotted in relation to (A) CR and (B) PD. As in Table 1, CR and PD are presented as z-scores following normalization that also took into account values from sated smokers and non-smokers. Table S1. Resting state functional connectivity (RSFC) differences among non-smokers, sated smokers, and abstinent smokers. Comparisons were performed using unpaired t-tests. ACC, anterior cingulate cortex; GP, globus pallidus; NAcc, nucleus accumbens; iPFC, inferior prefrontal cortex; mPFC, medial prefrontal cortex; sPFC, superior prefrontal cortex; SMA, supplementary motor area. Table S2. Top 5000 results from GWAS of c [file 12864_2017_4124_MOESM1_ESM.zip › Figure S2. Null QQs.tiff]

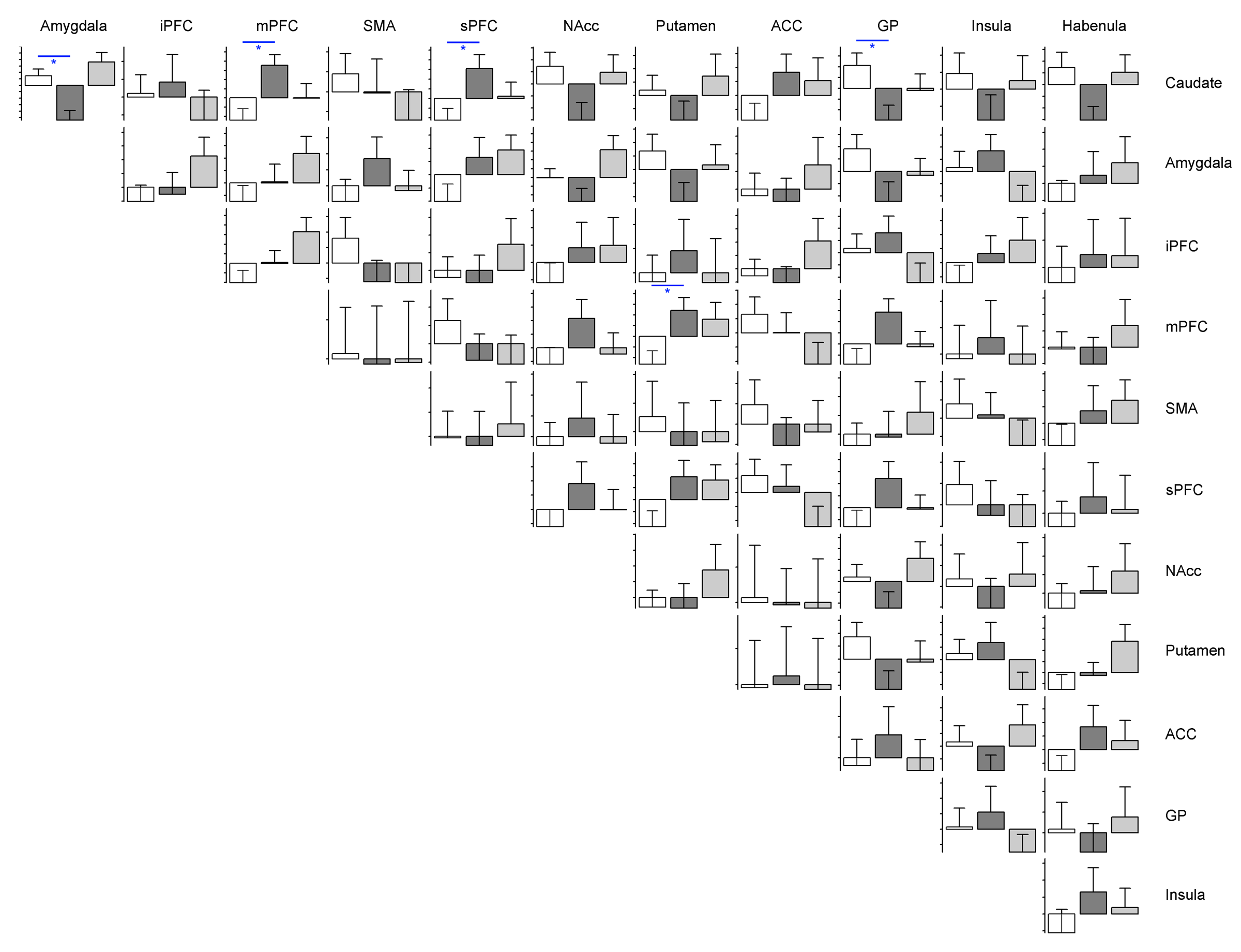

Supplement: Additional file 1: Figure S1. — Resting state functional connectivity (RSFC) among 12 brain regions. Rows and columns indicate the regions that comprise pairs for which RSFCs were computed. White, dark-gray, and light-gray bars represent average z-scores from magnetic resonance imaging measurements for non-smokers, sated smokers, and abstinent smokers, respectively, with standard errors marked above. All tickmarks are spaced 0.1 apart. Asterisks highlight differences that are significant at the 0.01 level. ACC, anterior cingulate cortex; GP, globus pallidus; NAcc, nucleus accumbens; iPFC, inferior prefrontal cortex; mPFC, medial prefrontal cortex; sPFC, superior prefrontal cortex; SMA, supplementary motor area. Figure S2. Quantile-quantile plots of GWAS results. With adjustment for smoker status and population stratification, allelic dosages of germline variants were linearly regressed on (A) caudate activity during disappointment, and (B) putamen activity during reward. The negative logarithms (base 10) of observed P-values were plotted in relation to those of expected P-values. Figure S3. Abstinence-associated withdrawal severity versus caudate activity during reward (CR) and putamen activity during disappointment (PD). After smokers had refrained from smoking for at least 12 h, the severity of their withdrawal symptoms was assessed using the Shiffman-Jarvik Withdrawal Questionnaire (SJWQ) and plotted in relation to (A) CR and (B) PD. As in Table 1, CR and PD are presented as z-scores following normalization that also took into account values from sated smokers and non-smokers. Table S1. Resting state functional connectivity (RSFC) differences among non-smokers, sated smokers, and abstinent smokers. Comparisons were performed using unpaired t-tests. ACC, anterior cingulate cortex; GP, globus pallidus; NAcc, nucleus accumbens; iPFC, inferior prefrontal cortex; mPFC, medial prefrontal cortex; sPFC, superior prefrontal cortex; SMA, supplementary motor area. Table S2. Top 5000 results from GWAS of c [file 12864_2017_4124_MOESM1_ESM.zip › Figure S1. Comparisons of RSFC.tiff]
